# Scorpion Sheds ‘Tail’ to Escape: Consequences and Implications of Autotomy in Scorpions (Buthidae: *Ananteris*)

**DOI:** 10.1371/journal.pone.0116639

**Published:** 2015-01-28

**Authors:** Camilo I. Mattoni, Solimary García-Hernández, Ricardo Botero-Trujillo, José A. Ochoa, Andrés A. Ojanguren-Affilastro, Ricardo Pinto-da-Rocha, Lorenzo Prendini

**Affiliations:** 1 Universidad Nacional de Córdoba, Córdoba, Argentina; 2 Universidad Industrial de Santander, Bucaramanga, Colombia; 3 Museo Argentino de Ciencias Naturales “Bernardino Rivadavia”, Buenos Aires, Argentina; 4 Frankfurt Zoological Society, Cusco, Perú; 5 Pontificia Universidad Javeriana, Bogotá, Colombia; 6 Universidade de São Paulo, São Paulo, Brazil; 7 American Museum of Natural History, New York, New York, United States of America; Estacion Experimental de Zonas Áridas (CSIC), SPAIN

## Abstract

Autotomy, the voluntary shedding or detachment of a body part at a determined cleavage plane, is a common anti-predation defense mechanism in several animal taxa, including arthropods. Among arachnids, autotomy has been observed in harvestmen, mites, and spiders, always involving the loss of legs. Autotomy of the opisthosoma (abdomen) was recently reported in a single species of the Neotropical buthid scorpion genus *Ananteris* Thorell, 1891, but few details were revealed. Based on observations in the field and laboratory, examination of material in museum collections, and scanning electron microscopy, we document autotomy of the metasoma (the hind part of the opisthosoma, or ‘tail’) in fourteen species of *Ananteris*. Autotomy is more common in males than females, and has not been observed in juveniles. When the scorpion is held by the metasoma, it is voluntarily severed at the joints between metasomal segments I and II, II and III, or III and IV, allowing the scorpion to escape. After detachment, the severed metasoma moves (twitches) automatically, much like the severed tail of a lizard or the severed leg of a spider, and reacts to contact, even attempting to sting. The severed surface heals rapidly, scar tissue forming in five days. The lost metasomal segments and telson cannot be regenerated. Autotomy of the metasoma and telson results in permanent loss of the posterior part of the scorpion’s digestive system (the anus is situated posteriorly on metasomal segment V) and the ability to inject venom by stinging. After autotomy, scorpions do not defecate and can only capture small prey items. However, males can survive and mate successfully for up to eight months in the laboratory. In spite of diminished predation ability after autotomy, survival allows males to reproduce. Autotomy in *Ananteris* therefore appears to be an effective, adaptive, anti-predation escape mechanism.

## Introduction

Autotomy is the process by which some animals voluntarily shed or detach a body part, usually as an anti-predator defense mechanism. In order to be considered autotomy, the process of shedding or detachment must be provoked by external stimuli, achieved by an intrinsic mechanism, and mediated by the nervous system [1–3].

Autotomy occurs along permanent sites of weakness, cleavage planes that permit a clean break when a body part is detached, with anatomical features that minimize trauma and promote rapid sealing of the fluid compartment, leading to swift closure and healing of the wound [2, 4].

Among invertebrates, autotomy is characterized by limited loss of hemolymph from either the stump or discarded appendage [3]. The separation of an appendage from the body at a site of weakness, when pulled by an outside agent, has been termed autospasy by some authors (e.g. [5, 6]), and is here considered synonymous with autotomy.

The detached body part, e.g., the tail of lizards or the legs of arthropods [7, 8], may also act as a distraction, engaging the predator’s attention by spontaneously twitching, writhing or wriggling, while the animal escapes. The incidence of autotomy in natural populations may be determined by predation efficiency and intensity, anatomical mechanisms, microhabitat preference, sex and ontogenetic differences, intraspecific aggression and the use of other defense mechanisms [3, 8].

Autotomy of a body part is an effective anti-predator defense mechanism that evolved independently in various taxa [3]. The phenomenon has been recorded in cnidarians, annelids, molluscs, echinoderms, arthropods and vertebrates [3, 7, 9]. Among arthropods, autotomy has been reported in crustaceans, hexapods, chilopods and arachnids, always involving the loss of appendages, usually legs [10–14]. Among arachnids, autotomy of the legs has been observed in Opiliones (harvestmen), Acari (mites and ticks), and Araneae (spiders) [3, 5, 6, 15], but was not reported to occur in scorpions. Autotomy of the metasoma, the posterior part of the opisthosoma, or ‘tail’, was recently reported in several species of the Neotropical buthid scorpion genus *Ananteris* Thorell, 1891 [16–18], which currently comprises 79 species of small (9 to 42 mm in total adult length), cryptic, terrestrial scorpions inhabiting the tropical forests of northern South America, from Costa Rica to Argentina [19–26]. These reports represent the first cases of autotomy of opisthosoma (abdomen) in arthropods.

Based on observations in the field and laboratory, examination of material in museum collections, and scanning electron microscopy, we document autotomy of the metasoma in fourteen species of the genus, and provide behavioral observations with the following objectives: (1) to verify that metasomal autotomy in *Ananteris* is provoked by external stimuli, achieved by an intrinsic mechanism, and mediated by the nervous system; (2) to confirm that cleavage planes are present in the metasoma of *Ananteris*; (3) to assess whether the incidence of autotomy differs between the sexes; and (4) to provide data about post-autotomy behavior which may illuminate the functional significance of metasomal autotomy.

## Materials and Methods

### Collecting permits

Collecting permits were issued by the following agencies: Brazil, Ministério do Meio Ambiente, Instituto Chico Mendes de Conservação da Biodiversidade (ICMBio #17974-3), and Instituto Brasileiro do Meio Ambiente e dos Recursos Naturais Renováveis (IBAMA #10148-1); Bolivia, Ministerio de Medio Ambiente y Agua (MMAyA VMA-DGBAP #1319); Perú, Direccion General de Forestal y Fauna Silvestre (#002-2008-INRENA-IFFS-DCB); Ecuador, Ministerio de Ambiente (scientific research authorization #007-14 IC-FAU-DNB/MA); Argentina, Secretaría de Ambiente de la Provincia de Córdoba (#SECA01-524433053-908). Specimens from Colombia belong to registered biological collections and did not require of specific permissions for this study. Specimens from Venezuela are part of old collections for which no permissions could be traced. This study did not involve any endangered or protected species.

### Field observations of autotomy

**Figure 1:**
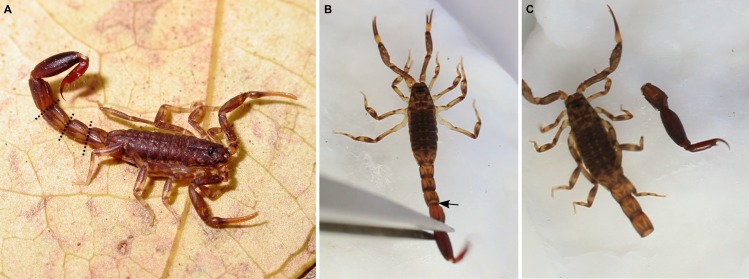
Autotomy in *Ananteris* Thorell, 1891 scorpions. A. *Ananteris balzani* Thorell, 1891, adult male from Serra das Araras Ecological Station, Mato Grosso State, Brazil. Dashed lines indicate autotomy cleavage planes between metasomal segments I-IV. B, C. Autotomy in *Ananteris solimariae* Botero-Trujillo & Flórez, 2011, adult male, video frames. B. Exact moment before autotomy, scorpion fighting to escape. Arrow indicates beginning of cleavage. C. Immediately after autotomy, detached tail twitching.

*Ananteris* specimens were collected in Argentina, Bolivia, Brazil, Colombia, Peru and Venezuela (details of collection localities in S1 Appendix) by turning stones during the day, or by ultraviolet (UV) light detection at night [27], using portable UV lamps, comprising mercury vapor tubes attached to a chromium reflector, and powered by a 12V, 7 Amp/hour battery, or Maglite flashlights modified with UV LED attachments. Autotomy was recorded when part of the metasoma was shed by an individual during collection. When autotomy occurred, the sex, age (adult or juvenile), and site of detachment (metasomal segments on either side of the cleavage plane, Fig. 1A), were noted.

### Museum records of autotomy

The scorpion collections of several museums were searched for *Ananteris* specimens that had undergone autotomy prior to collection (complete list of material examined in S1 Appendix). Autotomy was recorded as present when part of the metasoma (including telson) was missing, and a brown scar was evident at its severed stump (as *Ananteris* specimens become brittle after ethanol fixation, we could not be confident that autotomy had occurred if the metasoma was broken but no scar was evident). The species, sex, age, site of detachment, and presence of a scar, were noted for each specimen. The incidence of autotomy, i.e., the percentage of scorpions with part of the metasoma (including telson) missing and a scar present at the severed stump, in a single population was calculated for those species with more than ten specimens from a single collection locality. The results for different populations of the same species were not combined because it was assumed that the incidence of autotomy could vary among localities due to ecological factors (e.g., presence and abundance of predators). Differences among sexes and stages (adult males vs adult females vs immatures) were analyzed as 2×3 contingency tables with Fisher’s exact test in R v. 3.1.1 statistical package [28].

### Experimentally-induced autotomy

Twenty-five adult males and five adult females of *Ananteris solimariae* Botero-Trujillo & Flórez, 2011 were collected for experimentation from a population at Girón, Santander Department, Colombia. One of the females, gravid when captured, gave birth, and two second instar juveniles from her litter were also used in the experiments.

Scorpions were housed separately in plastic containers (7 cm diameter; 10 cm height) with moistened cotton as water supply and fed every two weeks with crickets, *Gryllodes sigillatus* Walker, 1869. After 7 to 10 days of acclimation, each adult scorpion was placed separately in a plastic container with a rough surface (humid cotton), and subjected to the following treatment, designed to demonstrate if detachment was provoked by external stimuli and achieved by an intrinsic mechanism. The metasoma was held with forceps on segments III, IV or V and gently pulled backwards to simulate capture by a predator, for no more than 30 seconds (sufficiently short duration to categorize the phenomenon as autotomy). The time to detachment of the metasomal segments was noted, if applicable. In order to avoid damaging the small second instar juveniles with the forceps, the posterior part of the metasoma was instead attached to sticky tape, which was pulled backwards with the forceps. Ten adult males were subjected to the same treatment, but using glass Petri dishes as a substrate, and the same specimens were held in the air for 30 seconds, without being allowed to contact any substrate. Experiments were filmed with a SONY Cyber-shot DSC-W35 camera and photographs taken with a Canon EOS Rebel T2i camera fitted with a 50 mm macro lens. The healing process of the wound was also documented, by photographing the development of scars on the severed stump of the metasoma during successive time intervals. The effect of anesthesia on autotomy was also investigated, to confirm whether autotomy is mediated by the nervous system, by placing another five adult male individuals of *A. solimariae* in a styrofoam box with ice, prior to manipulation of the metasoma. Differences between the experiments (adult males on rough substrate vs adult females on rough substrate; adult males on rough substrate vs adult males in Petri dishes; adult males on rough substrate vs adult males held in the air; adult males on rough substrate vs anesthetized adult males on rough substrate) were analyzed as 2×2 contingency tables with Fisher’s exact test in R v. 3.1.1 [28].

Additional experiments, identical to those performed on *A. solimariae*, were conducted with 92 live individuals (32 adult males, 32 adult females, 28 immatures) of ten other scorpion species in seven genera and three families: Bothriuridae Simon, 1880: *Bothriurus cordubensis* Acosta, 1995: 3 adult males, 2 adult females; *Bothriurus flavidus* Kraepelin, 1911: 2 adult males, 1 adult female, 3 immatures; *Brachistosternus ferrugineus* Thorell, 1876: 4 adult males, 5 adult females, 5 immatures; *Timogenes elegans* Mello-Leitão, 1931: 4 adult males, 1 adult female; *Timogenes dorbignyi* Guérin Méneville, 1843: 3 adult males, 1 adult female; *Urophonius brachycentrus* Thorell, 1876: 2 adult males, 4 adult females, 3 immatures; Buthidae C.L. Koch, 1837: *Tityus trivittatus* Kraepelin, 1898: 5 adult females; *Zabius fuscus* Thorell, 1876: 8 adult males, 6 adult females, 4 immatures; *Zabius birabeni* Mello-Leitão 1938: 4 adult males, 2 adult females; Hormuridae Laurie, 1896: *Opisthacanthus elatus* Gervais, 1844: 2 adult males, 5 adult females, 13 immatures. The bothriurid and buthid specimens were collected at several localities in Córdoba Province, Argentina, whereas the *O. elatus* specimens were collected in Santander Department, Colombia, in 2012.

### SEM of cleavage plane and scar

In order to determine whether a defined cleavage plane exists in the metasoma, six ethanol-preserved specimens of three buthid species (two adult specimens per species), *Ananteris balzani* Thorell, 1891, *Tityus uruguayensis* Borelli, 1901 and *Z. fuscus*, the last two species included for comparison with *A. balzani*, were manipulated with forceps to induce detachment of the metasoma between segments II and III, and III and IV. Scanning electron micrographs (SEM) were taken of the sites of detachment in these specimens, as well as of the scarred, post-autotomy metasomal segments of two *A. solimariae* specimens, with a Philips XL30 TMP SEM. Samples for SEM were dehydrated and coated with gold-palladium in a Thermo VG Scientific SC 7620 sputter coater.

### Post-autotomy behavior

The behavior of *A. solimariae* specimens post-autotomy was recorded in the laboratory and, when possible, compared with the behavior of intact (i.e., pre-autotomy) specimens. In order to assess the effect of losing part of the metasoma on male mating success, four mating trials were conducted with two adult females and four adult males, two with the metasoma intact and two without the last three metasomal segments. Each pair was placed in a mating arena (20 × 40 × 30 cm) with a substrate comprising soil, stones and pieces of tree bark from the collection locality. Mating behavior was observed and filmed under a 40 W red lamp. Two mating trials were conducted per female. The first two trials, involving males with an intact metasoma, were conducted when the females were gravid. The second and third trials, involving post-autotomy males, were conducted several months later, after both females had given birth and the juveniles had left their mothers.

## Results

### Field observations and museum records of autotomy

Evidence of autotomy was found in fourteen species of *Ananteris* (Table 1, S1 Appendix). In several species, part of the metasoma readily detached during capture in the field: holding the metasoma for a few seconds was sufficient to induce the scorpion to shed part of the metasoma, consistent with autotomy. In one case, involving an adult male *A. balzani* from Mato Grosso, Brazil, the detached metasoma writhed intensely, as if attempting to sting, for about one minute. Autotomy was not observed in *A. solimariae* specimens anesthetized with ice prior to manipulation, or when *A. balzani* and *A. solimariae* specimens were held by body parts other than the metasoma (i.e., the prosoma, mesosoma, pedipalps or legs). Other defense behaviors exhibited by some species at the time of collection included fleeing and tanatosis.

**Table 1:** Records of metasomal autotomy in scorpions of the genus *Ananteris* Thorell, 1891 (Buthidae).

				**Inter-segments where autotomy occurs**			
**Species**	**Males**	**Females**	**Observation**	**I**–**II**	**II**–**III**	**III**–**IV**	**Country: state, region or province**
*Ananteris arcadioi* Botero-Trujillo, 2008 *	2		F, S			1	Colombia: Meta
*Ananteris ashmolei* Lourenço, 1981	1		F			1	Ecuador: Napo
*Ananteris balzani* Thorell, 1891	7		F, S		2	5	Brazil: Mato Grosso, Mato Grosso do Sul, Minas Gerais, São Paulo
*Ananteris charlescorfieldi* Lourenço, 2001	2	1	F		1	2	Bolivia: Santa Cruz
*Ananteris columbiana* Lourenço, 1991	1	2	S		1	2	Colombia: Córdoba, Magdalena
*Ananteris dekeyseri* Lourenço, 1982	3		S			3	Brazil: Amazonas
*Ananteris dorae* Botero-Trujillo, 2008	1		S		1		Colombia: Nariño
*Ananteris ehrlichi* Lourenço, 1994		1	S		1		Colombia: Caquetá
*Ananteris solimariae* Botero-Trujillo & Flórez, 2011 *	23	1	F, L	2	4	16	Colombia: Santander
*Ananteris venezuelensis* González-Sponga, 1972		1	S			1	Venezuela: Bolívar
*Ananteris* sp. cf. *ehrlichi*	1		S		1		Colombia: Vaupes
*Ananteris* sp. 1	1		S		1		Colombia: Amazonas
*Ananteris* sp. 2	1		F			1	Perú: San Martín
*Ananteris* sp. 3	4	1	S		3	2	Venezuela: Bolívar
Total	**47**	**7**		**2**	**15**	**34**	

L = autotomy observed in the laboratory; F = autotomy observed in the field; S = collected with a well-developed scar on the severed stump of the metasoma.

* Some data about detached segments are missing.

Most cases of autotomy involved adult males (*n* = 23). The phenomenon was observed in only six adult females and not in immatures. The metasoma detached most frequently between segments III and IV (Table 1, *n* = 33), less often between II and III (Table 1, *n* = 15), and only twice between I and II (Table 1).

Incidence of autotomy in the field for five populations of four species was low, from 5.26 to 8.33% (Table 2). Only adult males were observed with part of the metasoma missing (10 out of 96). The metasoma was intact in all adult females (46) and immatures (12) observed. The frequency of autotomy was significantly greater among adult males than adult females and immatures across all species (Fisher’s exact test, *p* = 0.04605), but the difference was not significant within each species (*p* > 0.05). Autotomy affected 8.33 to 14.29% of the adult males in the population.

**Table 2:** Incidence of metasomal autotomy in wild populations of the scorpion genus *Ananteris* Thorell, 1891 (Buthidae).

				**Incidence (%)**	
**Species (population)**	**Adult Males**	**Adult Females**	**Immatures**	**Total**	**Adult Males**
*Ananteris arcadioi* Botero-Trujillo, 2008 (Villavicencio)	11 (1)	6	2	5.26	9.09
*Ananteris balzani* Thorell, 1891 (Pirassununga)	36 (3)	9	5	6	8.33
*Ananteris balzani* Thorell, 1891 (Serra das Araras)	11 (1)	7	2	5	9.09
*Ananteris solimariae* Botero-Trujillo & Flórez, 2011 (Girón)	10 (1)	7	0	5.88	10
*Ananteris* sp. 3 (Bolívar)	28 (4)	17	3	8.33	14.29
**Total**	96 (10)	46	12	6.49	10.42

Count of specimens collected with part of metasoma detached and with a well-developed scar on the severed stump (parentheses). No adult females or immatures were observed with a well-developed scar on the severed stump of the metasoma.

### Experimentally-induced autotomy

Under laboratory conditions, 22 of 25 adult males and one of five adult females of *A. solimariae* shed part of the metasoma after being held with forceps for 30 seconds or less, whereas three adult males, four adult females and both immatures did not. The frequency of autotomy was significantly greater among adult males than adult females (Fisher’s exact test, *p* = 0.005796).

Autotomy only occurred when scorpions were in contact with a rough substrate with their pedipalps and/or legs. Autotomy did not occur when scorpions were in contact with smooth, slippery surfaces (glass Petri dishes, *n* = 10), when held in the air (*n* = 10), or when anesthetized with ice prior to manipulation (*n* = 5). The frequency of autotomy was significantly greater among adult males on rough surfaces (22 out of 25) than adult males on Petri dishes (0 of 10), adult males held in the air (0 of 10) and anesthetized adult males (0 of 5) (Fisher’s exact test, *p* < 0.0001 in all cases).

Time to detachment of the metasomal segments, after contact with the forceps (Fig. 1B), varied from 0.29 to 12.99 seconds (median, 4.68 seconds). The metasoma detached most frequently between segments III and IV (*n* = 16) (Fig. 1C), less often between II and III (*n* = 4), and rarely between I and II (*n* = 2). Cleavage occurred between metasomal segments III and IV if the specimen was held with the forceps on segments IV, V, or on the telson, between segments II and III if held on segments III or IV (see S1 Movie), and between segments I and II if held on segment III. A lateral twisting motion of the metasomal segments anterior to the cleavage plane, immediately preceding detachment, was interpreted as part of the process of metasomal autotomy (see S2 Movie). Autotomy occurred rapidly, requiring minimal stimulus with the forceps, in some individuals whereas others only shed the metasoma after several attempts to escape (see S3 Movie).

The detached metasoma of some individuals carried part of the digestive tract of the preceding metasomal segments along with it (see Movies S1–S4).

**Figure 2:**
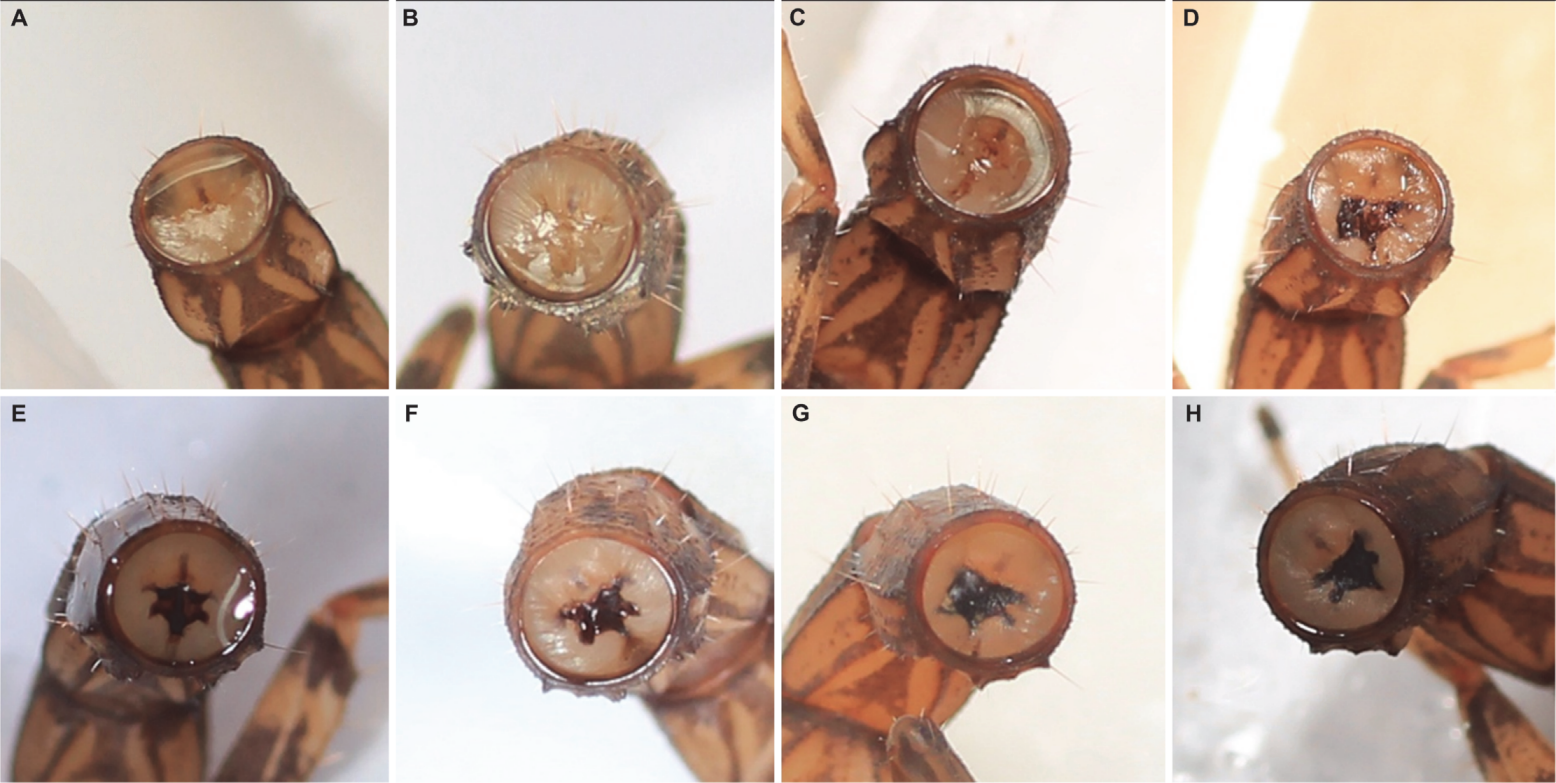
Post-autotomy healing of severed stump of metasomal segment of adult male *Ananteris solimariae* Botero-Trujillo & Flórez, 2011. A. One hour after autotomy, with drop of hemolymph. B. One day after, hemolymph loss continues. C. Two days after, hemolymph loss reduced, brown scar beginning to develop. D. Three days after, hemolymph loss reduced, scar developing. E. Four days after, scar almost completely developed. F. Five days after, no hemolymph loss, scar fully formed. G. Ten days after, scar darkened. H. Twenty-five days after, scar fully defined.

After autotomy, the detached metasoma writhed vigorously, as if attempting to sting, for some time (8.8–169.95 seconds; median, 49.32 seconds). If touched, the telson reacted to stimuli, attempting to sting in 47% (*n* = 19) of cases (see Movies S1 and S4). Immediately after autotomy, a small drop of hemolymph was evident at the site of detachment (Fig. 2A). Some loss of hemolymph occurred from the wound during subsequent days, but reduced with time, with no further loss after five days (Figs. 2B-F). The process of scar formation was rapid. One day after autotomy, a brown spot appeared medially, apparently produced by hemolymph coagulation, eventually darkening to form a blackish brown scar (Figs. 2G-H) which completely blocked the digestive system, preventing defecation.

Post-autotomy survival rate was high. All individuals were alive three weeks afterward and only one male (5%; *n* = 22) was dead by day 25. The remainder survived up to 8 months in the laboratory. No evidence of autotomy was observed in the other scorpion taxa tested.

### Post-autotomy behavior

**Figure 3:**
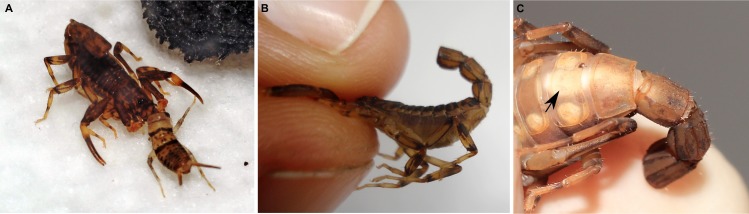
*Ananteris solimariae* Botero-Trujillo & Flórez, 2011, adult males, twenty-five days after autotomy. A. Feeding on cricket nymph. B. Attempting to sting, showing swollen opisthosoma. C. Accumulated excrement evident as white area inside opisthosoma (arrow).

Scorpions offered prey every two weeks after autotomy only fed on small crickets (up to 5 mm, adult males of *A. solimariae* range from 25–27 mm in total length, whereas adult females reach up to 34.32 mm in total length) using the pedipalps and chelicerae to grab and consume them alive (Fig. 3A). After 20–25 days, the opisthosoma of these individuals had become swollen due to the accumulation of excrement visible ventrally through the sternites in the posterior part of the mesosoma (Figs. 3B, C). In two cases, a second autotomy was observed. The first case was apparently spontaneous (no prey was present when it occurred, and the round, plastic container contained nothing which could have caused the specimen inside to become stuck) and occurred between metasomal segments I and II, both of which had remained (along with segment III) following detachment of segments IV and V, eight months earlier. In this case, autotomy was apparently induced by the internal pressure of the excrement because, without having been subjected to manipulation, metasomal segments II and III were found to have detached and a new scar developed at the severed stump on segment I. The detached segment was empty, and did not contain living tissue. The second case of autotomy, also between segments I and II, was induced by holding segment III (the segment that remained after the first autotomy) with forceps. In both cases, several drops of white excrement were expelled and a new scar developed at the end of the segment, several days after detachment.

After autotomy, scorpions attempted to use the remainder of the metasoma to sting prey and for defense, when captured, as if the metasoma and telson were fully intact (Figs. 3A, B). Attempts to attack prey of moderate to large size met without success and these individuals could only capture small prey with the pedipalps and chelicerae. Other behavior was similar to that observed prior to autotomy, except for a grooming behavior performed with the telson and posterior segments of the metasoma (lubricating these segments with fluid from the mouthparts, and using them to clean the body), which was no longer possible. The single adult female that underwent autotomy, was apparently not gravid.

The first two mating trials with post-autotomy males and gravid females terminated rapidly after female aggression led to stinging and cannibalization of the males. In contrast, two complete courtship rituals, both ending in successful sperm transfer, were observed with post-autotomy males and post-parturition females (see S5 Movie). At the onset of courtship, after making contact with the female, the male curved what remained of his metasoma, performing balanced movements from side to side. Afterward, the male grasped the female’s pedipalps with his, and guided the courtship ‘dance’, walking backwards. On several occasions, the couple interrupted the dance, and the male grasped the female chelicerae with his (cheliceral ‘kiss’; [29]), without releasing her pedipalps. After locating a suitable substrate (rock or dry leaf), the male deposited his spermatophore and moved slightly backwards, guiding the female over it. During the course of guiding the female over the spermatophore, which lasted 70 to 85 seconds, and while continuing to grasp her pedipalps, the male moved rhythmically, by rapidly vibrating his pedipalps and gently pushing the female every three seconds, touched the ventral surface of the female’s prosoma and genital operculum with his first legs (‘rubbing with legs’; [29]), and touched her chelicerae and anterior carapace margin with his chelicerae. When sperm transfer was complete, the male disengaged the pedipalps and ran away, after limited aggression by the female. Neither female ate the spermatophore. Except for the first contact with the female, neither male made any further attempt to use the severed stump of the metasoma. As a complete courtship ritual with the intact males was not observed, and courtship behavior has not been previously described in any *Ananteri*s species, it is impossible to know whether male *Ananteri*s perform ‘clubbing’ (striking the partner with the metasoma while the sting is tucked away [29]) or the ‘sexual sting’ (male punctures the female’s body with his aculeus [29]).

### SEM of cleavage plane and scar

**Figure 4:**
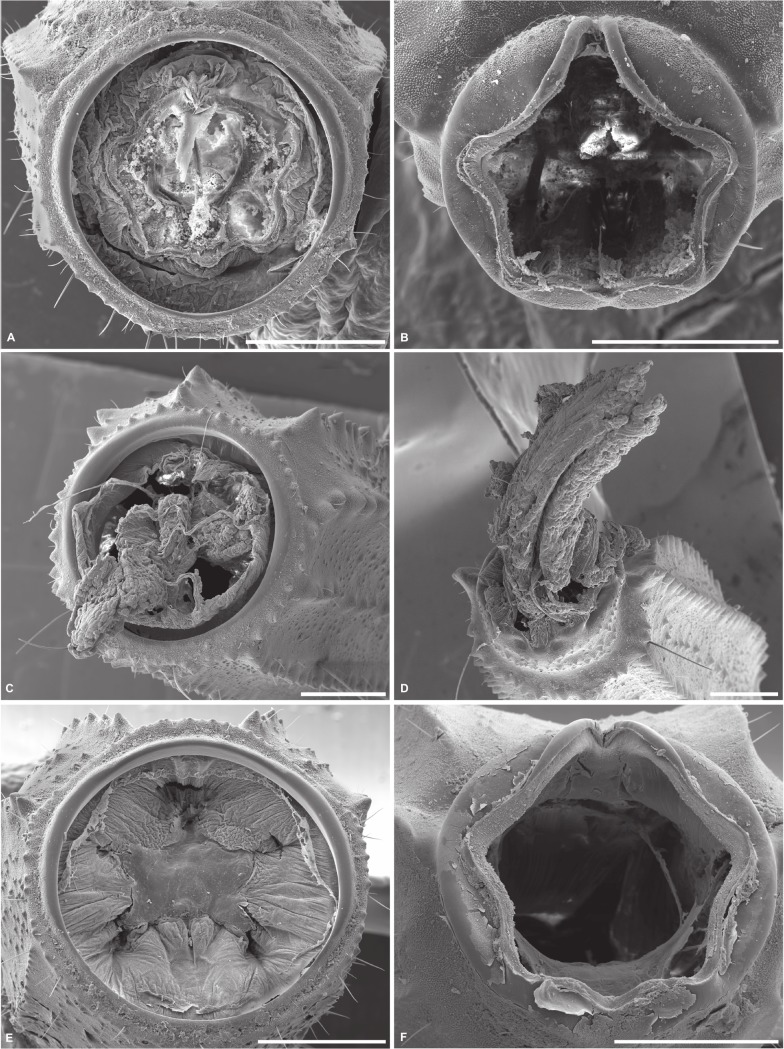
Scanning electron micrographs of cleavage planes on severed stumps of metasomal segments of selected buthid scorpions. A, B. *Ananteris balzani* Thorell, 1891, segments III, posterior end (A) and IV, anterior end (B), manipulated with forceps to induce detachment. C, D. *Zabius fuscus* (Thorell, 1876), segments II, posterior end (C) and III, anterior end (D), detached with forceps. E, F. *Ananteris solimariae* Botero-Trujillo & Flórez, 2011, segments III, posterior end (E) and IV, anterior end (F), post-autotomy. Scale bars = 0.5 mm.

Scanning electron micrographs of the metasomal segments of ethanol-preserved specimens of *A. balzani* revealed a cleavage plane between segments II and III and between segments III and IV (Figs. 4A, B). The severed surfaces exhibited sharp edges (indicative of a clean break) in the tegument of the intersegmental membrane, almost without a trace of the severed metasomal muscle or digestive system. In contrast, similar tests on other buthid scorpion species revealed ragged edges in the tegument of the intersegmental membrane, with part of the muscles and digestive system protruding (Figs. 4C, D). The wound on the severed stump of a post-autotomy *A. solimariae* displayed a large star-shaped scar closing the posterior end of the segment (Fig. 4E), whereas the severed surfaces exhibited sharp edges, without any structures protruding (Fig. 4F).

## Discussion

Metasomal detachment in *Ananteris* meets the criteria for defensive autotomy [2, 3]. Detachment is provoked by external stimuli and achieved by an intrinsic mechanism: in order to occur, the metasoma must be grasped by a potential predator (simulated by holding it with forceps in the experiments presented here) and the scorpion must pull forward with its pedipalps and/or legs contacting a rough surface, allowing the metasomal segments to separate along permanent sites of weakness (cleavage planes). The lateral twisting motion of the metasomal segments anterior to the cleavage plane, and the fact that autotomy did not occur in anesthetized specimens, suggest that the process is mediated and controlled by the nervous system.

Autotomy in *Ananteris* provides a mechanism for escape from predation. Movements of the detached metasomal segments are presumably controlled by the last or second-last of the four neural ganglia, situated anteriorly in each of the first four metasomal segments. The fourth ganglion has a pair of nerves, extending posteriorly into two branches, one innervating metasomal segment V and the other innervating the telson [30], which provide these detached segments with the ability to react to stimuli, increasing the effectiveness of distraction.

There are several costs associated with autotomy in *Ananteris*. A potentially significant cost, post-autotomy, is the loss of part of the digestive system, including the posterior part of the mesenteron (midgut or middle intestine), the entire proctodeum (hindgut or posterior intestine, contained in metasomal segment V), and the anus, which opens at the posteroventral end of segment V, in the intersegmental membrane preceding the telson [30]. The only visible effect of losing part of the digestive system appears to be the accumulation of excrement inside the mesosoma, caused by the inability to defecate. This is the first report, to our knowledge, of a case in which autotomy prevents defecation. The ability of scorpions to excrete very little waste, consisting mostly of insoluble nitrogenous compounds [31], may permit their survival despite this handicap. Furthermore, it may be possible to spontaneously release accumulated excrement during at least one additional autotomy event, as described above. The observed cases of a second autotomy, taken together with the finding that most cases of autotomy occurred between metasomal segments III and IV, suggest that autotomy of more posterior metasomal segments (i.e., between segments III and IV rather than II and III or I and II) is selectively advantageous for several reasons. Detachment of the metasoma between segments III and IV appears to leave more intestinal space for accumulation of excrement, and may provide a chance to release accumulated excrement during a second or third autotomy event, as well as an additional chance of escape from predators. Individuals that undergo autotomy between segments III and IV may survive longer, allowing more opportunity to increase their reproductive success, than those which undergo autotomy between segments II and III or I and II, in part because of the potential for additional autotomy events.

Loss of the telson, which bears the venom gland and aculeus (sting), negatively affects the scorpion’s ability to catch larger prey or to sting potential predators, and considerably reduces its defense capabilities, but post-autotomy scorpions can still capture and feed successfully on smaller prey. A similar observation has been reported in crabs with autotomy of the chelipedes [32].

Another possible cost of autotomy could be the loss of metasomal photoreceptors, which have been identified in a few scorpion species to date [33, 34]. These photoreceptors differ from the median and lateral ocelli in their sensitivity to wavelengths of light [35] and may assist with phototaxic behavior [36]. Their presence in *Ananteris* has not yet been determined, however.

Although autotomy results in permanent loss of the posterior part of the digestive system and the ability to inject venom by stinging, among other possible costs, *Ananteris* scorpions are able to survive and mate successfully. A complete sequence of courtship behavior has not yet been described in any species of *Ananteris*, hence is impossible to know whether male *Ananteris* perform ‘clubbing’ (striking the partner with the metasoma while the sting is tucked away [29]) or the ‘sexual sting’ (male punctures the female’s body with his aculeus [29]). Nevertheless, two males with an incomplete metasoma successfully completed sperm transfer. As males are able to survive several months after autotomy, they have time to increase their reproductive success by mating with multiple females for as long as they remain alive. In the absence of life history data (including natural lifespan) for any species of *Ananteris*, it is impossible to know whether autotomy actually shortens the lifespan but even if that were the case, autotomy could potentially increase male survival and reproductive success, by allowing males to escape from predators and mate on more occasions. Autotomy may be adaptive because it allows *Ananteris* scorpions to survive predation.

Although we have data for few species and populations, the incidence of autotomy in the field was low (up to 8.33%, increasing to 14.29% when only adult males are considered) and restricted to adult males. The higher incidence of autotomy in adult males may be explained by the difference in breeding behavior between the sexes. Male scorpions are more vagile, wandering in search of females during the breeding season, placing them at greater risk of predation [15]. The difference in the incidence of autotomy between the sexes may also be attributed to differences in survival and reproductive success. In most scorpion species, adult males live no more than one or two reproductive seasons, whereas adult females live much longer [15]. Autotomy may reduce the space available for a female’s embryos (and hence the size of her litter) due to an accumulation of excrement in the opisthosoma, resulting in a decrease in reproductive success, compared with escaping from a predator intact. Furthermore, females could be less predisposed to undergo autotomy to avoid losing the sting, which greatly enhances predation ability. Females need more food for embryonic development during gestation.

Including *Ananteris mauryi* Lourenço, 1982 [18], at least fifteen species of *Ananteris* exhibit autotomy, which may prove to be synapomorphic for the genus, but further observations on the remaining species and/or a phylogenetic hypothesis confirming their monophyly are needed to confirm this hypothesis. Research into the costs and benefits of metasomal autotomy in *Ananteris* are also needed, to explore how trade-offs may have influenced its evolution, and the occurrence of autotomy should be investigated in other scorpion taxa.

## Supporting Information

S1 Appendix *Ananteris* Thorell, 1891 material examined with evidence of autotomy, e.g., part of metasoma missing and with a developed scar (S), or autotomy observed in the field (F) or laboratory (L). (DOC)

S1 Movie Complete sequence of metasomal autotomy in a male *Ananteris solimariae* Botero-Trujillo & Flórez, 2011 scorpion. The metasoma (“tail”) was held with forceps and gently pulled backwards to simulate capture by a predator. After autotomy, the detached metasoma writhed vigorously, and the telson (segment posterior to fifth metasomal segment, containing “sting”) reacted to stimuli when touched.
(AVI)

S2 Movie Slow motion (10% of real time) sequence of the moment of metasomal autotomy in a male *Ananteris solimariae* Botero-Trujillo & Flórez, 2011 scorpion, illustrating lateral twisting motion of the metasomal segments anterior to the cleavage plane, immediately preceding detachment of the posterior metasomal segments. (AVI)

S3 Movie Complete sequences of metasomal autotomy in three male *Ananteris solimariae* Botero-Trujillo & Flórez, 2011 scorpions, illustrating differences in duration. (AVI)

S4 Movie Complete sequence of metasomal autotomy in a female *Ananteris solimariae* Botero-Trujillo & Flórez, 2011 scorpion, illustrating white excrement and part of digestive system. Detached metasoma writhed vigorously post-autotomy, but the telson (segment posterior to fifth metasomal segment, containing “sting”) did not react to stimuli when touched.
(AVI)

S5 Movie Final stage of mating in a pair of *Ananteris solimariae* Botero-Trujillo & Flórez, 2011 scorpions, including a post-autotomy male, missing the posterior three metasomal segments. Sperm transfer occurred successfully and an empty spermatophore is evident at the end of the sequence.
(AVI)
