# Supplementary material for: Scorpion Sheds ‘Tail’ to Escape: Consequences and Implications of Autotomy in Scorpions (Buthidae: Ananteris)
Source: PLoS One. 2015 Jan 28;10(1):e0116639. doi: 10.1371/journal.pone.0116639 (PMC4309614; doi:10.1371/journal.pone.0116639)
Supplement: S1 Appendix — (DOC) [file pone.0116639.s001.doc]

**Appendix S1.** *Ananteris* Thorell, 1891 material examined with evidence of autotomy, e.g., part of metasoma missing and with a developed scar (S), or autotomy observed in the field (F) or laboratory (L).

Materials are deposited in the following collections: American Museum of Natural History, New York, USA (AMNH); Instituto de Investigación de Recursos Biológicos Alexander von Humboldt, Villa de Leyva, Colombia (IAvH); Instituto de Ciencias Naturales, Universidad Nacional de Colombia, Bogotá, Colombia (ICN-UN); Laboratorio de Biología Reproductiva y Evolución, Universidad de Córdoba, Argentina (LBRE); Museo Argentino de Ciencias Naturales “Bernardino Rivadavia”, Buenos Aires, Argentina (MACN); Museo de Historia Natural, Facultad de Ciencias Biológicas, Universidad Nacional de San Antonio Abad del Cusco, Peru (MHNC); Museo de Historia Natural La Salle, Caracas, Venezuela (MHNLS); Museo Javeriano de Historia Natural “Lorenzo Uribe S. J.”, Pontificia Universidad Javeriana, Bogotá, Colombia (MPUJ); Museu de Zoologia, Universidade de São Paulo, Brazil (MZSP); Glauco Machado personal collection, São Paulo, Brazil (GM); Solimary García Hernández personal collection, Santander, Colombia (SGH).

***Ananteris arcadioi* Botero-Trujillo, 2008:** 1 male (S) (AMNH), Colombia: Meta, between Villavicencio and Restrepo, Hacienda “Con Esto Tengo”, 04°11'51"N 73°35'37"W, 7 September 2008, R. Botero-Trujillo & J.A. Ochoa.

***Ananteris ashmolei* Lourenço, 1981:** 1 male (F) (presumably, escaped after autotomy, leaving detached metasoma) (MACN), Ecuador: Napo, Río Hollín, road to Santo Domingo, 5 km SE from Archidona, Sacha Wuagra, 0º57'19.60"S 77º44'51.50"W, 660 m, 15-16 March 2014, R. Botero-Trujillo, J. A. Ochoa.

***Ananteris balzani* Thorell, 1891:** 1 male (F) (MZSP), Brazil: Mato Grosso: Rodovia BR-163, limit MT/MS, 17º30'46.3"S 54º44'23.1"W, 21 October 2010, C. Mattoni, A. Ojanguren-Affilastro, R. Almeida & J.A. Ochoa; 1 male (S) (MZSP 36929), Brazil: Mato Grosso: Parque Estadual Serra das Araras: Porto Estrella, 15º39'05.6"S 57º12'52.2"W, 22 –24 October 2010, C. Mattoni, A. Ojanguren-Affilastro, R. Almeida & J.A. Ochoa; 1 male (F) (MACN), Brazil: Parque Nacional da Serra da Bodoquena: Bonito area, 20º45'14.9"S 56º48'14.3"W, 19 October 2010, C. Mattoni, J.A. Ochoa, R. Almeida & A. Ojanguren-Affilastro; 1 male (S) (AMNH), Brazil: Minas Gerais: Cavernas do Peruaçu National Park, 15º7' 25.79"S 44º14'28"W, 25 –26 January 2007, C. Mattoni, R. Pinto-da-Rocha & H. Yamaguti; 3 males (S) (GM), Brazil: São Paulo: Pirassununga, Campus USP, 21º56'45"S 47º27'54"W, pitfall traps, 17 September 2000–30 March 2001.

***Ananteris charlescorfieldi* Lourenço, 2001:** 2 males (F), 1 female (F) (presumably, escaped after autotomy, leaving detached metasoma) (MZSP), Bolivia: Santa Cruz: Buena Vista, 17°29'57.4"S 63°39'4.4"W, 2 December 2010, R. Pinto-da-Rocha, A. Benedeti, A. Sarabia & J.A. Ochoa.

***Ananteris columbiana* Lourenço, 1991:** 1 female (S) (ICN-As 585), Colombia: Córdoba: Pueblo Nuevo, Hacienda Toronto, 08°24'N 75°17'W, 7 –13 June 2004, J.D. Lynch; 1 male (S) (MPUJ-SCO-364), 1 female (S) (MPUJ-SCO-363), Colombia: Magdalena: Santa Marta, Sierra Nevada de Santa Marta Natural National Park, 11°15'N 74°12'W, December 2006, J.A. Noriega.

***Ananteris dekeyseri* Lourenço, 1982:** 3 males (S) (MZSP), Brazil: Amazonas: Manaus, Reserva Ducke, 2°55'S 59°59'W, 16–18 November 2010, J.A. Ochoa & A. Pepato.

***Ananteris dorae* Botero-Trujillo, 2008:** 1 male (S) (ICN-As 816), Colombia: Nariño: Barbacoas, Altaquer, Río Ñambi Natural Reserve, 01°17'N 78°04'W, 1440 m, 13 October 2009, E. Flórez & D. Luna.

***Ananteris ehrlichi* Lourenço, 1994:** 1 female (S) (ICN-As 579), Colombia: Caquetá: La Montañita, Santuario Las Iglesias, Itarca, 01°29'N 75°26'W, 330 m, 25 April 2004, M. Agudelo.

***Ananteris* aff. *ehrlichi*:** 1 male (S) (ICN-As 575), Colombia: Vaupes: Taraira, Estación Biológica Caparú,01º04'31.6"S 69º30'49.5"W, 196 m, 1 April 2004, J. Pinzón.

***Ananteris solimariae* Botero-Trujillo & Flórez, 2011:** 1 male (F) (ICN-As 779), 22 males and 1 female (L) (SGH, LBRE, MACN), Colombia: Santander: Girón, Quintas del Llanito, 07°04'15"N 73°10'23"W, 703 m, June 2009, S. García Hernández.

***Ananteris venezuelensis* González-Sponga, 1972:** 1 female (S) (AMNH), Venezuela: Bolivar: Gran Sabana, Mt. Roraima, 05º11'N 60º44'W, December 1927.

***Ananteris* indet. (sp. 1):** 1 male (S) (IAvH), Colombia: Amazonas: Amacayacu Natural National Park: Mata Mata, 03°23'S 70°06'W, 150 m, 17–19 June 2000, A. Parente.

***Ananteris* indet. (sp. 2):** 1 male (F) (MHNC), Perú: San Martín: Tarapoto, close to Pongo de Cayñarachi, 6º19'36"S 76º17'16"W, 11 January 2008, J.A. Ochoa.

***Ananteris* indet. (sp. 3):** 3 males (S) (AMNH), 1 male (S) (MHNLS), 1 female (F) (MHNC), Venezuela: Bolivar: Gran Sabana, Campamento Uruyen, 5º41'08"N 62º25'43"W, 30 July 2009.
